# Evaluation of an augmented reality platform for austere surgical telementoring: a randomized controlled crossover study in cricothyroidotomies

**DOI:** 10.1038/s41746-020-0284-9

**Published:** 2020-05-21

**Authors:** Edgar Rojas-Muñoz, Chengyuan Lin, Natalia Sanchez-Tamayo, Maria Eugenia Cabrera, Daniel Andersen, Voicu Popescu, Juan Antonio Barragan, Ben Zarzaur, Patrick Murphy, Kathryn Anderson, Thomas Douglas, Clare Griffis, Jessica McKee, Andrew W. Kirkpatrick, Juan P. Wachs

**Affiliations:** 1 School of Industrial Engineering, Purdue University, West Lafayette, IN USA; 2 Department of Computer Science, Purdue University, West Lafayette, IN USA; 3 Paul G. Allen School of Computer Science and Engineering, University of Washington, Seattle, WA USA; 4 Department of Surgery, School of Medicine, Indiana University, Indianapolis, IN USA; 5 Naval Medical Center Portsmouth, Portsmouth, VA USA; 6 Department of Surgery, and the Regional Trauma Services, University of Calgary, Calgary, AB Canada; 7 Department of Critical Care Medicine, University of Calgary, Calgary, AB Canada; 8 Canadian Forces Medical Services, Ottawa, ON Canada

**Keywords:** Health care, Computer science

## Abstract

Telementoring platforms can help transfer surgical expertise remotely. However, most telementoring platforms are not designed to assist in austere, pre-hospital settings. This paper evaluates the system for telementoring with augmented reality (STAR), a portable and self-contained telementoring platform based on an augmented reality head-mounted display (ARHMD). The system is designed to assist in austere scenarios: a stabilized first-person view of the operating field is sent to a remote expert, who creates surgical instructions that a local first responder wearing the ARHMD can visualize as three-dimensional models projected onto the patient’s body. Our hypothesis evaluated whether remote guidance with STAR could lead to performing a surgical procedure better, as opposed to remote audio-only guidance. Remote expert surgeons guided first responders through training cricothyroidotomies in a simulated austere scenario, and on-site surgeons evaluated the participants using standardized evaluation tools. The evaluation comprehended completion time and technique performance of specific cricothyroidotomy steps. The analyses were also performed considering the participants’ years of experience as first responders, and their experience performing cricothyroidotomies. A linear mixed model analysis showed that using STAR was associated with higher procedural and non-procedural scores, and overall better performance. Additionally, a binary logistic regression analysis showed that using STAR was associated to safer and more successful executions of cricothyroidotomies. This work demonstrates that remote mentors can use STAR to provide first responders with guidance and surgical knowledge, and represents a first step towards the adoption of ARHMDs to convey clinical expertise remotely in austere scenarios.

## Introduction

Timely and adequate treatment is an essential factor in the survival of critically injured patients^1^, particularly in the pre-hospital setting^2,3^. In such situations, treatment administered by medical personnel specialized in immediate emergency services (i.e. first responders)^4^ is crucial for casualty survival^5,6^. Although first responders are trained to deliver pre-hospital care for a number of conditions, telementoring is being explored as a method of increasing the breadth, depth, and effectiveness of pre-hospital care by delivering expert assistance remotely. Telementoring platforms are called upon to bridge the geographic distance between experts and novice or less experienced care providers^7,8^. In doing so, patient care can be improved, first responders’ education can be enhanced, and patient access to experienced care can be increased^9^. For example, in simulated damage control laparotomy scenarios, military medics’ have shown improved procedural execution of out of scope interventions, with improved self-confidence when they are mentored by an expert^10,11^. Other examples include the use of mobile and tablet devices by members of Canadian Armed Forces and the Israeli Defense Forces to receive support from remote mentors during simulated pre-hospital hemorrhage control scenarios^12–15^.

Two main approaches for enhancing mentor–mentee communication during telementoring procedures have been explored: telestrators and augmented reality (AR). In the telestrator approach, a remote mentor annotates a live video of the mentee’s operating field using lines and icons that encode surgical instructions^16^. These annotations are visualized by the local mentee on a nearby display. Albeit effective, telestrator approaches require mentees to constantly shift focus away from the operating field to visualize the annotations on the nearby display and to remap them to the actual operating field, which can lead to extra cognitive loads and errors^17,18^. AR has been explored as an alternative to telestrators, with good results^19,20^. In AR telementoring, three-dimensional (3D) computer-generated objects are superimposed into the field of view of the mentee, in real time, which avoids focus shifts^21^. In most of these AR-based approaches, tablets between the patient and the mentee are used to display the medical guidance, placed in a fixed position in the operating field^19,20^.

Nonetheless, most telestrator-based or AR tablet-based telementoring platforms are not designed to provide point of injury (POI) care. For example, such platforms are neither self-contained nor portable: they require multiple pieces of hardware to operate (e.g. external cameras, screens, computers, brackets), which would introduce undesired encumbrance and delays in a POI setting^22^. Additionally, the setup of most telestrator-based or AR tablet-based telementoring platforms can limit the surgeons’ free selections of motions^23^, can degrade depth perception due to loss of stereopsis^24^, and can lead to mentor high cognitive loads and even simulator sickness if not paired with image stabilization routines^10,25^. Because of these shortcomings, the reliance of such platforms in two-way audio communication is prevalent, making it the most common telementoring approach^26^.

To address the shortcomings of telestrator-based or AR tablet-based telementoring platforms, this paper evaluates, in a simulated austere scenario, the next-generation system for telementoring with augmented reality (STAR). STAR is a self-contained and portable surgical telementoring platform that leverages an augmented reality head-mounted display (ARHMD). The platform can be used to provide medical assistance remotely by combining first-person view stabilization routines together with the ability to project virtual medical instructions onto the patients’ body. Our experiment tests the hypothesis of whether the STAR platform could help mentees perform an emergency cricothyroidotomy procedure with higher scores, as assessed by experienced surgeons, compared to audio-only telementoring. Participants were evaluated in terms of their performance, non-procedural skills, and overall execution. The audio-only condition was selected as our control condition because it represents the bare minimum support a first responder can receive during a POI scenario.

Our experimental setup evaluated participants performing cricothyroidotomies using two telementoring conditions: Audio, in which participants received remote expert guidance through a speakerphone; and STAR, in which participants wore STAR’s ARHMD-based platform in addition to receiving audio guidance through a speakerphone. The participants performed the cricothyriodotomies in a simulated austere environment that included smoke and loud noises of gunshots and explotions. Two remote attending general surgeons mentored the participants through the steps of procedure, while two other experienced surgeons evaluated the participants’ performance on-site. The on-site evaluators assessed the participants’ performance using five main metrics: Emergency Cricothyroidotomy Performance (ECP) scores, Global Rating Scale (GRS), Evaluator’s Overall Rating (EOR), Critical Criteria (CC), and Completion Time. Three of these scores included both subscores describing specific performance criteria (ECP-1 to ECP-10; GRS-1 to GRS-6; CC-1 to CC-3) and a comprehensive scores describing overall performance (ECP-T, GRS-T, and CC-T). A within-subject statistical analysis based on a linear mixed regression model was run to compare both conditions (Audio and STAR) based on the performance scores obtained by the participants. Finally, the participants evaluated the telementoring conditions (e.g. ease of use, generated frustration) through a post-experiment questionnaire.

## Results

### Participants’ demographics

Twenty first responders (17 males, 3 females; age 26.2 ± 7.4) performed two cricothyroidotomies, for a total of 40 repetitions (20 per condition). The analyses were performed over 19 participants (Overall Population group), as the data from one participant were discarded due to a logistical error (the participant performed the procedure without receiving guidance). Participants reported between 6 months and 15 years of experience as first responders. Based on this information, a subgroup that included participants with fewer than 10 years of experience as first responders was evaluated (Low First Responder Experience, *N* = 16). Additionally, participants reported having performed between 0 and 7 cricothyroidotomies as part of their training. Based on this information, a second subgroup that included participants with fewer than three cricothyroidotomies in their prior training was evaluated (Low Cric Experience *N* = 12). The two groups had a 65% overlap. Table 1 reports the scores and *p* values obtained for all the metrics, for all comparison groups.

**Table 1:** Averaged results for the different comprehensive scores for the Overall Population and both the experience-based subgroups.

Comprehensive score	Overall Population *n* = 19			Low First Responder Experience *n* = 16			Low Cricothyroidotomy Experience *n* = 12		
	STAR^a^	Audio^a^	*P* values	STAR^a^	Audio^a^	*P* values	STAR^a^	Audio^a^	*P* values
ECP-T^b^	3.38 (0.45)	2.99 (0.79)	0.01*	3.35 (0.46)	2.83 (0.75)	0.01*	3.20 (0.45)	2.73 (0.74)	0.03*
GRS-T^b^	3.82 (0.81)	3.37 (1.21)	0.05*	3.68 (0.79)	3.10 (1.13)	0.05*	3.54 (0.82)	2.95 (1.09)	0.10
EOR^c^	80.84 (10.04)	73.94 (17.52)	0.02*	79.25 (10.18)	70.12 (16.35)	0.01*	77.50 (9.88)	73.5 (12.12)	0.04*
CC-T^d^	0.89 (0.32)	0.63 (0.50)	0.04*	0.88 (0.34)	0.56 (0.51)	0.04*	0.83 (0.39)	0.58 (0.51)	0.16
Completion Time^e^	274.79 (91.86)	272.11 (108.67)	0.94	287.19 (95.25)	285.00 (113.86)	0.95	281.08 (105.87)	298.08 (117.55)	0.66

^a^Mean (standard deviation).

^b^1–5 score.

^c^0–100 score.

^d^0/1 score.

^e^Time in seconds.

*P* values with an asterisk (*) represent a significant difference between the telementoring conditions.

### Evaluating the participants’ performance, non-procedural skills, and overall execution

All comparison groups obtained higher ECP scores when using STAR. The comprehensive ECP-T score was significantly higher when using STAR than when using Audio for the Overall Population, the Low First Responder Experience, and the Low Cric Experience groups (*p* = 0.01, *p* = 0.01, and *p* = 0.03, respectively). The summarized ECP subscores are shown in Table 2. Finally, the procedure completion time did not reveal statistically significant differences between the conditions.

**Table 2:** Averaged results for the different criteria of the Emergency Cricothyroidotomy Performance scores for the Overall Population and both the experience-based subgroups.

Emergency cricothyroidotomy procedure evaluation form criteria	Overall Population *n* = 19			Low First Responder Experience *n* = 16			Low Cricothyroidotomy Experience *n* = 12		
	STAR^a,b^	Audio^a,b^	*P* values	STAR^a,b^	Audio^a,b^	*P* values	STAR^a,b^	Audio^a,b^	*P* values
ECP-1	3.58 (0.61)	3.26 (0.87)	0.24	3.50 (0.63)	3.13 (0.89)	0.56	3.33 (0.65)	3.00 (0.95)	0.42
ECP-2	3.42 (0.77)	3.11 (1.10)	0.26	3.31 (0.79)	2.94 (1.12)	0.70	3.25 (0.87)	2.83 (1.27)	0.42
ECP-3	2.89 (0.94)	2.84 (1.01)	0.76	2.81 (0.91)	2.63 (0.96)	0.79	2.50 (0.90)	2.58 (1.00)	0.90
ECP-4	2.74 (1.28)	2.53 (1.58)	0.52	3.00 (0.89)	2.25 (1.57)	0.52	2.50 (1.17)	2.42 (1.44)	0.78
ECP-5	3.42 (0.61)	3.05 (0.91)	0.11	3.31 (0.60)	2.88 (0.89)	0.83	3.33 (0.65)	2.92 (0.79)	0.15
ECP-6	3.79 (0.92)	3.37 (1.50)	0.30	3.75 (1.00)	3.25 (1.61)	0.55	3.67 (1.15)	3.00 (1.81)	0.27
ECP-7	3.89 (0.46)	3.63 (1.01)	0.29	3.88 (0.50)	3.56 (1.09)	0.53	3.83 (0.58)	3.50 (1.24)	0.46
ECP-8	3.58 (0.61)	2.74 (1.48)	0.05*	3.56 (0.63)	2.63 (1.54)	0.84	3.42 (0.67)	2.50 (1.45)	0.13
ECP-9	3.53 (0.51)	2.68 (1.25)	0.02*	3.44 (0.51)	2.50 (1.26)	0.85	3.42 (0.51)	2.25 (1.29)	0.06
ECP-10	3.00 (0.82)	2.74 (1.05)	0.24	2.94 (0.85)	2.56 (1.03)	0.36	2.75 (0.75)	2.33 (0.98)	0.21

^a^Mean (standard deviation).

^b^1–5 score.

*P* values with an asterisk (*) represent a significant difference between the telementoring conditions (*p* ≤ 0.05).

Additionally, all comparison groups obtained higher GRS scores when using STAR. The comprehensive GRS-T score was significantly higher when using STAR than when using Audio for the Overall Population and the Low First Responder Experience groups (*p* = 0.05 and *p* = 0.05, respectively). The summarized GRS subscores are shown in Table 3. Furthermore, all comparison groups obtained higher EOR scores when using STAR. The EOR scores of all groups were significantly higher when using STAR than when using Audio for the Overall Population, the Low First Responder Experience, and the Low Cric Experience groups (*p* = 0.02, *p* = 0.01 and *p* = 0.04, respectively).

**Table 3:** Averaged results for the different criteria of the Global Rating Scale metric for the Overall Population and both the experience-based subgroups.

Global Rating Scale criteria	Overall Population *n* = 19			Low First Responder Experience *n* = 16			Low Cricothyroidotomy Experience *n* = 12		
	STAR^a,b^	Audio^a,b^	*P* values	STAR^a,b^	Audio^a,b^	*P* values	STAR^a,b^	Audio^a,b^	*P* values
GRS-1	3.95 (1.03)	3.63 (1.07)	0.19	3.75 (1.00)	3.44 (1.03)	0.27	3.58 (1.00)	3.25 (0.97)	0.28
GRS-2	3.84 (0.90)	3.47 (1.22)	0.21	3.63 (0.81)	3.19 (1.11)	0.21	3.50 (0.90)	3.08 (1.08)	0.31
GRS-3	3.84 (0.90)	3.42 (1.26)	0.18	3.69 (0.87)	3.13 (1.15)	0.17	3.50 (0.90)	3.00 (1.13)	0.26
GRS-4	3.84 (0.76)	3.21 (1.36)	0.08	3.75 (0.77)	2.94 (1.29)	0.06	3.58 (0.79)	2.75 (1.29)	0.10
GRS-5	3.68 (0.95)	3.26 (1.28)	0.21	3.56 (0.96)	3.00 (1.21)	0.19	3.50 (1.00)	2.83 (1.19)	0.18
GRS-6	3.79 (0.85)	3.21 (1.32)	0.11	3.69 (0.87)	2.94 (1.24)	0.07	3.58 (0.90)	2.83 (1.19)	0.13

^a^Mean (standard deviation).

^b^1–5 score.

No statistical significance (*p* ≤ 0.05) was found between the telementoring conditions.

Finally, all comparison groups obtained higher CC scores when using STAR. The comprehensive CC-T score was significantly higher when using STAR than when using Audio for the Overall Population and the Low First Responder Experience groups (*p* = 0.04 and *p* = 0.04, respectively). The summarized CC subscores are shown in Table 4.

**Table 4:** Averaged results for the different criteria of the Critical Criteria metric for the Overall Population and both the experience-based subgroups.

Cricothyroidotomy Critical Criteria	Overall Population *n* = 19			Low First Responder Experience *n* = 16			Low Cricothyroidotomy Experience *n* = 12		
	STAR^a,b^	Audio^a,b^	*P* values	STAR^a,b^	Audio^a,b^	*P* values	STAR^a,b^	Audio^a,b^	*P* values
CC-1	1.00 (0.00)	0.89 (0.32)	0.98	1.00 (0.00)	0.88 (0.34)	0.98	1.00 (0.00)	0.92 (0.29)	0.98
CC-2	1.00 (0.00)	0.89 (0.32)	0.97	1.00 (0.00)	0.88 (0.34)	0.97	1.00 (0.00)	0.83 (0.39)	0.98
CC-3	0.89 (0.32)	0.68 (0.48)	0.06	0.88 (0.34)	0.63 (0.50)	0.07	0.83 (0.39)	0.67 (0.49)	0.27

^a^Mean (standard deviation).

^b^0/1 score.

No statistical significance (*p* ≤ 0.05) was found between the telementoring conditions.

## Discussion

STAR is self-contained, portable and does not require the setup of additional cameras or extensive calibration routines to operate. By using the device’s onboard camera to transmit a view of the operating field, our system eliminated the need of external cameras that could encumber the medics who work in austere settings^23^. Our platform leverages an ARHMD device instead of other AR-based approaches relying on tablets under the rationale of avoiding extra encumbrance and time delays^19,20^. A previous work in the STAR platform analyzed the workspace efficiency of using ARHMD technologies instead of tablets^23^. The aforementioned work found that health practitioners would have collided or modified their motions as they performed a practice procedure had a tablet device been there. Additionally, tablet-based telementoring systems introduce an additional setup stage to place the tablet in a fixed location in the operating field. Such additional setup stage would introduce undesired encumbrance and delays that must be avoided when providing immediate care^27^.

Nonetheless, the same previous work commented on how portable telementoring can only be achieved if the view of the operating field provided to the mentor is obtained using a wearable camera instead of an external camera (i.e. first-person view instead of third-person view). However, unstabilized first-person views provide a discontinuous visualization that is not suitable for proper mentor situational awareness^28^. To address this issue, our ARHMD platform integrated first-person view stabilization routines at the mentor site to correct for jitter and sudden head motions introduced by first-person visualizations. Combining these stabilization routines with the device’s portability and the ability to project surgical guidance onto the patient’s body results in a platform that is more suitable for austere, pre-hospital scenarios.

Our experiment tested the hypothesis of whether the STAR platform could help mentees perform an emergency cricothyroidotomy procedure with higher performance scores, as assessed by experienced surgeons, compared to audio-only telementoring. Since both conditions included audio communication, this experimental setup directly evaluated the effect of the capabilities of our ARHMD telementoring platform on the mentees’ performance (i.e. first-person view visualization of the operating field, and graphical annotations in 3D visualized directly over the patient’s body). The cricothyroidotomy procedure was selected because it is part of the corpsmen medical training and must be performed at POI when necessary^29^, making it adequate to evaluate our portable telementoring system.

Randomization methods were employed to reduce the number of confounding variables in our study. For example, switching the mentors/evaluators after four repetitions (every two participants) guaranteed that the mentors and evaluators would get exposure to both telementoring conditions, preventing biases related to only mentoring/evaluating the same condition throughout the experiment. Additionally, learning effect biases related to participants always starting the experiment in the same telementoring condition were reduced by randomizing the starting condition of each participant and running two trails in parallel. Finally, all participants completed an initial briefing regarding the experiment logistics. Likewise, the mentors and the evaluators were given an initial briefing regarding the steps to mentor/evaluate to keep the instructions and assessments as consistent as possible.

Overall, positive results favor telementoring with STAR over telementoring with Audio, both for the Overall Population group and the lower-experience subgroups. These subgroups represented the populations that would benefit the most from a telementoring experience due to their relative lower surgical expertise and were considered a placeholder for first responders in a POI scenario requiring assistance. These subgroups received lower scores in all metrics, which was expected due to the lower experience of their members.

The linear mixed model analyses showed that using STAR was associated with higher procedural outcomes, as represented by the higher ECP-T scores. Specifically, ECP-8 and ECP-9 also reported a statistical significance, as assessed with the proportional odds model. A fine level of visual detail was required for both steps to be considered well executed: the amount of air inserted in the cuff needed to be carefully assessed; otherwise, the correct placement on the cannula through the cricothyroid membrane could be compromised. The remote mentors in the STAR condition were able to visualize the operating field as the mentees performed the procedure, which allowed them to assess whether the steps were being performed correctly^30^. The visual feedback allowed the mentors to perform four corrections per participant on average (e.g. “You are not done yet; you need to check for bilateral breath sounds”). The visual feedback also allowed the participants to ask for instructions and confirmations an average of five times per procedure (e.g. “Should I make the incision longer?”). This type of feedback was not possible in the Audio condition, as mentors only relied on the mentees’ verbal confirmation to provide their feedback. Additionally, participants in the STAR condition received guidance for 1 min and 47 s on average, as opposed to only 57 s on average for participants in the Audio condition. These mentoring times represented 39% and 21% of the total task completion time, respectively. These values reveal that participants received remote guidance for almost double the time when they were in the STAR condition, which could have been one of the reasons of their higher ECP-T score.

Moreover, the mentors were able to guide the mentees better through the procedure using the AR annotations offered by the telementoring platform. The remote mentors created nine annotations per participant on average. The use of the annotations can be divided into three situations: (1) demonstrating which surgical tools to use (e.g. placing the icon of a scalpel to represent “Incise here”); (2) locating anatomical structures (e.g. drawing circles to indicate the location of the cricothyroid membrane); and (3) showing the location and length of incisions (e.g. drawing a vertical line over a section of the cricothyroid membrane). By creating these annotations, the mentors were able to convey more guidance, a possible reason of the increased performance when participants used the STAR condition.

On the other hand, the other ECP criteria did not show significant difference between the conditions. A possible reason for this finding is that the first responders, even those of low expertise, had the necessary knowledge to perform some steps of a cricothyroidotomy without requiring assistance. Examples of such steps include stabilizing the larynx and cutting through the cricothyroid membrane. Another possible reason is that the cricothyroidotomies were performed in a patient simulator, which could have reduced the participants’ stress levels and mitigated the type of complications that could arise while performing the procedure. We hypothesize that the telementoring capabilities of our system will be particularly useful when complications arise.

The linear mixed model analyses also showed that using STAR was associated with higher non-procedural outcomes, as represented by the higher GRS-T and EOR scores. This shows that participants benefitted from receiving additional guidance with STAR. For example, the remote mentors were able to prevent incorrect instrument usage and to point out when the wrong instrument was being used. According to the EOR criteria, the evaluators considered that the participants were able to perform the exposure with minimal difficulty in an expeditious fashion when using the STAR condition. Contrarily, the evaluators considered that the participants needed to review the procedure when using the Audio condition. This can be attributed to the capabilities of our telementoring platform. For example, providing the mentors with a first-person visualization allowed them to recognize and correct mentees as they performed the procedure. Additionally, the AR annotations conveyed mentees with more surgical guidance, such as length of incisions and anatomical landmarks to avoid.

The binary logistic regression analyses also showed that using STAR was associated with overall better performances, as represented by the higher CC-T scores. Specifically, the CC-3 revealed that evaluators considered the executions to be significantly safer during the STAR condition, as assessed with the proportional odds model. This can be attributed both to the constant visual assessment by the remote mentors, and to the augmented visual feedback received by the mentees, which illustrated eloquently how to perform the incisions without compromising patient safety.

Furthermore, the results of the questionnaires revealed that participants from all groups considered that STAR provided more information to complete the procedure, and the information was more helpful than the one received through audio. Additionally, participants from all groups felt that STAR reduced the time they took to complete the procedure. On the other hand, participants from the Overall Population and Low First Responder Experience groups mentioned that STAR caused some frustration. Nonetheless, participants in the Low Cric Experience group found Audio to be more frustrating. With respect to the ease to follow the instructions, the Overall Population group found instructions were harder to follow in the STAR condition. However, participants in the Low First Responder Experience group did not find difference between the conditions, and participants in the Low Cric Experience group found instructions were harder to follow in the Audio condition.

This study can be expanded in several ways. While the experimental design simulated an austere environment, real conditions experienced by first responders in the battlefield cannot be fully replicated (e.g. very high levels of stress, divided attention to maintain individual safety). Our work points at the suitability of using ARHMD-based telementoring platforms in austere scenarios, but validations in contexts closer to POI scenarios are required. Moreover, technical improvements need to be performed over the system. Specifically, the system’s stabilization routines make assumptions about the overall shape of the operating field. For example, the system represented the patient simulator’s neck as a flat surface. These assumptions should be revisited when dealing with complex and dynamic environments. Additionally, our system can be expanded to transmit body signals such as blood pressure and oxygen saturation, indicators that would be useful for prolonged care during triage^31^. Finally, other surgical procedures should be included to test the system’s generalization capabilities.

Technical limitations are also imposed by the selected ARHMD device. For example, the system lost its spatial tracking several times due to the smoke in the room. This is a technical limitation of the Microsoft HoloLens: the system relies on infrared and color cameras to obtain its location with respect to the environment, which were affected by the dense smoke conditions. The first-person stabilization was prone to annotation misalignments when the spatial tracking was lost. During our experiment, this was easily corrected by manually activating the system’s re-stabilization routines. However, a fallback mechanism needs to be defined and implemented to re-stabilize the system autonomously. Another technical limitation arises when this ARHMD device is used under direct harsh sunlight. For example, the brightness of these environments will make the AR annotations to look dim, and the onboard computer of the device tends to overheat due to being exposed to direct sunlight. Finally, the impact of “focal rivalry” needs to be analyzed in these contexts^32^. This effect refers to misalignments between the real scene and the AR annotations introduced by the focal length of ARHMDs (e.g. when tracing an incision line, the person wearing the ARHMD might see the AR annotation in a slightly different location than the one in which the mentor positioned it). We hypothesize that this could be tackled by fading the AR annotations over time, and by revising how the AR annotations are projected onto the scene.

The legal ramifications of telementoring technologies need to be determined before they can be integrated into real-world scenarios. For example, aspects such as who should be responsible in case of a system malfunction or a medical malpractice remain unaddressed. Insurance companies and hospital should also be consulted to define a model of integration of ARHMD telementoring platforms into medical curricula. Also, patients need to consent on receiving treatment from the remote specialist. Finally, an international medical authority should oversee and provide legal protection against sources of potential liability such as potential loss of patient privacy, insecure patient information, among others^33^.

In conclusion, our results revealed that our ARHMD-based platform allowed participants undergoing harsh field conditions (e.g. simulated gunshot and helicopter noises, low visibility due to smoke) to receive higher performance and non-procedural scores while performing cricothyroidotomies, as assessed by expert evaluators. Our study hints that telementoring capabilities of ARHMD-based platforms can be integrated to audio-based communication to improve the amount of medical guidance that first responders receive from remote experts during austere situations. Overall, this study positions ARHMD-based telementoring as a promising option for providing assistance in austere, POI scenarios in the military.

## Methods

### Concept

**Fig. 1:**
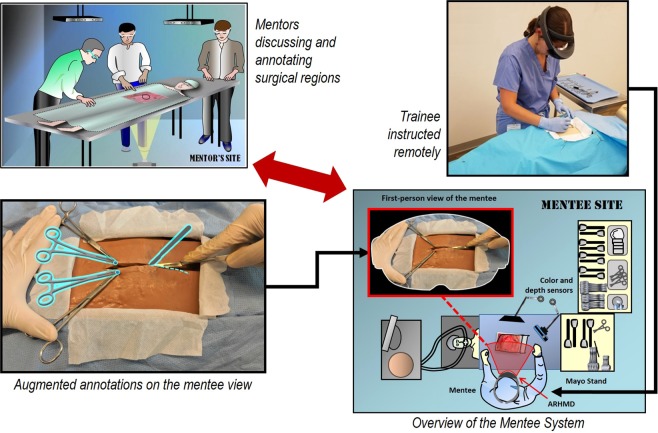
Schematic of the STAR platform. By integrating image stabilization routines with the ability to project mentor-authored surgical instructions onto the operating field, this portable system is designed to convey clinical expertise in austere scenarios. The trainee showcased gave written informed consent to have their photo used for this image.

STAR is a surgical telementoring platform that uses AR technology to display graphical surgical instructions authored by a remote mentor directly onto the field of view of a mentee. Figure 1 depicts a schematic of the STAR platform. At the operating site, a mentee wears our ARHMD system to record a first-person view of the operating field, which is sent to the mentor. In a previous work we identified that unstabilized first-person views acquired from an ARHMD’s onboard camera provided a discontinuous visualization that was not suitable for proper mentor situational awareness^23^. To address this issue, our platform integrated first-person view stabilization routines at the mentor site^34^. The approach receives the first-person video feed from the operating field, stabilizes it, and displays it on a large-scale interactive screen. These image stabilization routines correct the jitter and sudden head motions introduced by first-person visualizations^28^. The mentor creates annotations representing surgical instructions over this stabilized video feed using touch interactions (e.g. drawings incision lines, illustrates the placement of instruments), which are sent to the ARHMD at the mentee site. The mentee then visualizes these annotations, projected in 3D onto the patient’s body thanks to the ARHMD. This last step is a result of the ARHMD’s geometry acquisition routines, which maps the 2D-authored mentor instructions into 3D representations for stereo visualization^35^.

**Fig. 2:**
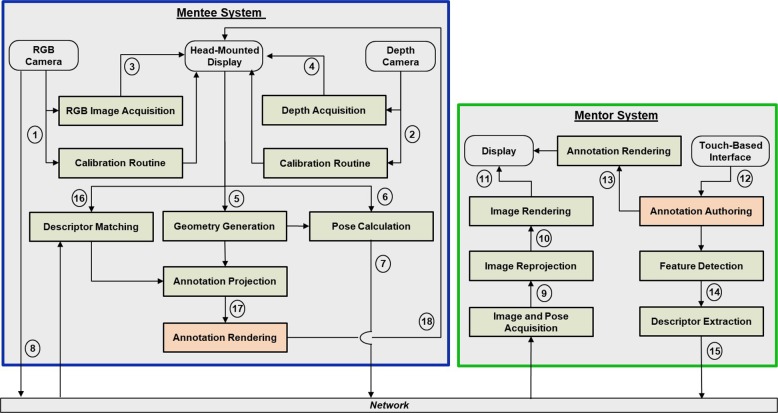
Architecture of the STAR platform. The view of the local mentee’s operating field is transferred over a network connection. After applying image stabilization routines, a remote mentor creates surgical instructions over this view, which are transferred to the mentee. The mentor-authored annotations can be projected onto the patient’s body at the correct position and depth thanks to the geometric reconstruction of the operating field created by the ARHMD.

Figure 2 presents the architecture of the STAR platform. First, the Mentee System undergoes initial calibration routines to align the ARHMD view with the operating field (1 and 2). The device’s onboard cameras (3 and 4) then acquire the color (RGB) and depth views of the operating field. The ARHMD generates an approximation of the geometry of the operating field based on the RGB and depth images (5). This geometry, along with internal sensors, is then used to calculate the ARHMD’s pose (6) with respect to the operating field. The pose and the RGB image are sent to the Mentor System using an internet connection (7 and 8). Once received by the Mentor System, stabilization routines that include aligning the image based on the received pose (9) and rendering the RGB image at the correct position (10) are applied to provide the mentor with a continuous visualization of the mentee’s operating field (11). The mentor can then use touch interactions (12) to create, modify, and visualize (13) annotations representing surgical instructions. The descriptors of these annotations, e.g. type, position, rotation, are extracted (14) and transmitted to the Mentee System (15). These descriptors are received and identified by the Mentee System (16) and their position with respect to geometry of the operating field is calculated (17). The projected annotations are finally rendered as virtual objects, visualized by the ARHMD at the correct position and depth (18).

The systems connected to the internet using WebRTC, a video transmission protocol that adjusts the video quality automatically based on a network’s bandwidth (similar to the protocol used by Skype™). On the mentor site, the system connected to the internet using the hospital’s Wi-Fi network. On the mentee site, an LTE cellular connection was used instead to simulate an austere condition with no Wi-Fi networks available. Once connected, video of 640 × 480 quality at 30 frames-per-second was streamed, with an average video latency of 1 s (including latency introduced by the image stabilization routines), and average AR annotation latency of less than 50 ms. The system requires a one-time purchase of an ARHMD device (below $3000) and a computer with touch-display based capabilities (below $1000 range). We use the Microsoft HoloLens (Microsoft HoloLens, Microsoft Corporation, Washington, USA; Windows 10 RS4 Build 17134) as our ARHMD. Finally, our research team created two standalone apps: the Mentee System App running in the ARHMD and the Mentor System App running in a Windows 10 computer (version 10.0.17763 or equivalent). These apps were programmed in Unity (version 2017.4.3f1) and compiled in Visual Studio 2017 (version 15.9.9 or later). The Mixed Reality Toolkit (https://github.com/Microsoft/MixedRealityToolkit-Unity; build 2017.4.0.0) was used for the AR capabilities of the Mentee System, and both the Mentor System and Mentee System use the HoloPoseWebRTC library (https://github.com/DanAndersen/HoloPoseWebRtc, commit 30651138c9) to access the WebRTC routines. These apps are hosted in GitHub and can be installed following an installation guide (Mentor System App: www.github.com/edkazar/MentorSystemUWPWebRTC/releases; Mentee System App: www.github.com/practisebody/STAR/releases).

### Experimental apparatus

Ethical approval (IRB #1705019165) was obtained from Purdue University and the Naval Medical Center Portsmouth (NMCP), and written participant consent was acquired for each participant. Every participant was briefed on the logistics of the experiment before starting and was able to withdraw from the experiment at any point. A randomized, controlled crossover experiment was performed to evaluate the STAR platform in a simulated austere environment. Two remote attending general surgeons mentored the participants who performed an emergency cricothyroidotomy procedure on a patient simulator (SimMan 3G, Laerdal, Stavanger, Norway). One of the remote mentors reported prior experience using telementoring platforms to instruct surgery residents and medical students through training leg fasciotomies. Each participant performed two cricothyroidotomies, in random order using a random number generator, in the following conditions: Audio, the control condition in which participants received remote guidance through a speakerphone; and STAR, the experimental condition in which participants wore STAR’s ARHMD-based platform to visualize the expert-authored surgical instructions, and received audio guidance through a speakerphone.

The experiment was conducted in four rooms at two separate medical facilities. Two rooms were located at a Level-1 trauma center at Indiana University’s School of Medicine (IUSM; Indiana, USA) and two rooms located at NMCP (Virginia, USA). The two rooms at IUSM were used as mentor stations: one room for the STAR condition and one room for the Audio condition. An attending surgeon was stationed in each mentor room to provide expert guidance in the performance of the cricothyroidotomy, using the room’s telementoring condition. Both mentor stations were equipped with a conference speakerphone (Konftel 300Mx, Konftel AB, Umeå, Sweden) to communicate with the mentees. Additionally, the mentor station for the STAR condition included a large-scale interactive screen (Aquos Board PN-L603B, Sharp Electronics, Osaka, Japan) that allow the mentor to see the mentees’ operating field and to author the surgical instructions.

Subsequently, two rooms located at NMCP were used as mentee stations, one for the STAR condition and one for the Audio condition. The mentee stations simulated an austere indoor environment: loud background sounds such as gunshots and helicopter engines were introduced, and smoke was blown into the room through the ventilation system to simulate low visibility due to explosions. Each mentee station was equipped with a cell phone (iPhone 6, Apple, California, USA) connected to a speakerphone (ZoeeTree S1, Shenzhen Zhiyi Technology Co., Shenzhen, China) to provide two-way audio communication with the mentors. The mentee rooms were equipped with a tripod-mounted camera (PTZ Pro 2, Logitech, Lausanne, Switzerland), which is not part of the telementoring platforms, and was used to record the participants for the purpose of the experiment. In addition, the mentee station for the STAR condition included the ARHMD, which was worn by the mentee. Finally, one expert evaluator was located in each of the mentee stations to assess the participants’ performance using an emergency cricothyroidotomy evaluation form.

### Participants

US Navy corpsmen were recruited as participants. This population was selected as an adequate placeholder for a frontline medic administering POI care in an austere scenario. No restrictions were established with respect to the participants’ years of experience, or with respect to the participants’ training level in cricothyroidotomies. The Low First Responder Experience and Low Cric Experience subgroups were defined to analyze the effectiveness of telementoring with respect to the participants’ level of experience.

### Creating the emergency cricothyroidotomy procedure evaluation form

On-site experts evaluated the participants’ performance using our emergency cricothyroidotomy evaluation form (Supplementary Methods 1). This evaluation form was created by combining two standard forms used to evaluate cricothyroidotomy procedures: the DA FORM 7595-2-10 from the U.S. Army Training and Doctrine Command^36^ and the “Emergency Surgical Airway Using the Cric-Key” skill sheet from the TCCC Handbook^37^. These forms are standard military assessments for evaluating the performance of an emergency cricothyroidotomy, and they include aspects such as landmark identification, incision performance, and proper patient ventilation, among others. The forms were combined because some of the evaluation aspects present in one form were not present in the other one. Criteria related to the isolation of body substances were not included due to patient simulator limitations.

As previously mentioned, our evaluation form comprised five main scores to evaluate the participants: ECP scores, GRS, EOR, CC and procedure completion time, measured in seconds. The ECP are 10 evaluation scores (ECP-1 to ECP-10) describing the performance of cricothyroidotomies in a comprehensive way. Originally, the ECP scores evaluated participants using a PASS/FAIL score^36^. However, based on the feedback obtained from expert evaluators after performing a pilot of our experiment, each ECP scores was changed to a five-level Likert scale (from 1 = really bad to 5 = really good; based on Melchiors et al.^38^). This change was performed because the expert evaluators considered that the original PASS/FAIL criteria did not provide them with enough resolution to evaluate the participants’ performance. Afterwards, each participant’s ECP scores were summarized into an overall score (ECP-T), calculated as the average of all the other ECP scores. The ECP-T score was calculated as an average to be consistent with range of the other ECP scores (1–5). We introduced this score as a comprehensive summary of the entire execution of the procedure, since none of the original evaluation forms included a score to quantify overall performance^36,37^. In general, each ECP score was assigned following: 1 = the step was not performed; 2 = the step was performed with difficulties and incorrectly; 3 = the step was performed without difficulties, but incorrectly; 4 = the step was performed correctly and but with difficulties; 5 = the step was performed correctly and without difficulties. The ECP scores evaluate:

- ECP-1: Correctly identified and palpated key surface landmarks on the anterior neck and the cricothyroid membrane.
- ECP-2: While stabilizing the larynx, made a vertical incision through the skin directly over the cricothyroid membrane.
- ECP-3: While continuing to stabilize the larynx, used tool or fingers to expose the cricothyroid membrane.
- ECP-4: Used the scalpel to make a horizontal incision through the cricothyroid membrane.
- ECP-5: Inserted the Crickit and Melker cannula through the cricothyroid membrane directed distally towards the lungs until the flange contacted the skin of the neck.
- ECP-6: Verbalized feeling for tracheal rings while inserting the Cric-Key
- ECP-7: Removed the Cric-Key, leaving the Melker cannula in place.
- ECP-8: Inflated the cuff of the Melker cannula with 10 ml of air.
- ECP-9: Checked for air exchange and verified placement of the tube by assessing for bilateral rise and fall of the chest.
- ECP-10: If air exchange was adequate, secured the Melker cannula in place.

Additionally, the GRS consisted of five-level Likert scale questions assessing non-procedural aspects such as knowledge of the procedure and instrument handling^39^. The GRS included six criteria (GRS-1 to GRS-6), which were summarized into a comprehensive score (GRS-T) that assessed the non-procedural aspects of the procedure, and was calculated as the average of all the other GRS criteria. In general, each ECP score was assigned following: 1 = very poor performance of evaluated criterion; 2 = poor performance of evaluated criterion; 3 = competent performance of evaluated criterion; 4 = good performance of evaluated criterion; 5 = superior performance of evaluated criterion. The GRS scores evaluate:

- GRS-1: preparation for procedure
- GRS-2: respect for tissue
- GRS-3: time and motion
- GRS-4: instrument handling
- GRS-5: flow of procedure
- GRS-6: knowledge of procedure

Furthermore, the EOR used a 0–100 score to evaluate the participants’ overall performance. The approach to assign the EOR scores is explained: a score below 60 represents that the participant is not ready to perform the procedure. A score between 60 and 69 represents that the participant can perform the procedure guided by an expert, but not when left alone. A score between 70 and 79 represents that the participant could perform the procedure alone after quickly revisiting a text or guide. A score between 80 and 89 represents that the participant can perform the procedure alone with minimal difficulties. Lastly, a score above 90 represents that the participants is excellent at performing the procedure.

Moreover, the Tactical Combat Casualty Care Handbook’s Critical Criteria (CC) are 0/1 scores evaluating the procedure in an overall manner: a score of zero in any of these represented an unsuccessful cricothyroidotomy. The three CC scores (CC-1 to CC-3) were summarized into a comprehensive score (CC-T) that represented whether the procedure was successfully performed. This CC-T score was calculated via the truth-functional operator of logical conjunction: the CC-T score took a value of 1 (true) if and only if all the other CC criteria were also 1, and a value of 0 (false) otherwise. The CC scores evaluate:

- CC-1: Obtained a patent airway with the emergency surgical airway.
- CC-2: Identified the location of the cricothyroid membrane.
- CC-3: Performed procedure in a manner that was safe to the casualty.

Finally, participants filled a questionnaire after completing each cricothyroidotomy. The questionnaire evaluated the telementoring conditions in terms of ease of use, quality and quantity of conveyed guidance, generated frustration, and time taken to complete the procedure.

### Randomization

Prior to the experiment, the mentors were instructed in the use of both telementoring conditions. Additionally, they were given a standard set of instructions to guide the first responders through the procedure. Likewise, the local evaluators were instructed on the use of their evaluation sheet prior to the experiment. During this briefing, the evaluators came with a consensus of what would they consider as correct or incorrect performance for each criterion on the evaluation sheet. The evaluators did not interact between each other after the briefing.

All enrolled participants were given an introduction to the study, which included an overview of the cricothyroidotomy procedure, of the patient simulator, and of the functionality of our ARHMD-based system. Participants were asked to wear the device and familiarize themselves with its adequate positioning to be able to see sample 3D world annotations. This final step was performed to reduce biases related to possible prior telementoring experiences the first responders may have had. Prior to the study, participants completed a questionnaire regarding demographics, cricothyroidotomy experience, and number of years of experience as first responders. After receiving the briefing and responding to the questionnaires, participants were grouped into pairs in a first-come, first-served basis. Using a random number generator, the participants in the pair were randomly assigned to start in either the STAR or the Audio condition. Upon completion of one repetition of the procedure, the participants switched between telementoring conditions to perform a second repetition. This was performed to ensure the participants had equal chances of starting the experiment in either condition. Every four cricothyroidotomies (two pairs of participants) were performed, the mentors switched between the Audio and STAR telementoring rooms, and the evaluators switched between the Audio and STAR participant rooms. This was performed to prevent biases introduced by having the mentors and the evaluators in the same experimental condition during the entire study.

### Statistical analysis

A within-subject statistical analysis was run to compare both conditions. The null hypothesis for all comparisons was that both conditions (Audio and STAR) will lead participants to comparable performance scores for all the comprehensive metrics (ECP-T, GRS-T, CC-T, EOR, Completion Time). The telementoring conditions were treated as independent variables, while the aforementioned metrics were treated as dependent variables. The data’s normality assumption was checked with the Shapiro–Wilk test^40^. In addition, the Levine’s test was run to assess the data’s equal variance assumption, revealing no need for a data transformation^41^. Afterwards, two different analyses were run depending on the type of data. For continuous responses (ECP-T, GRS-T, EOR, and Completion Time), a linear mixed model was run^42^, as the variability introduced into the model by the different mentor–evaluator pairs needed to be considered. The linear mixed regression model for the analysis is of the form:

$${\mathrm {Response}} = \beta _0 + \beta _1I_{\left\{ {\mathrm {STAR}} \right\}} + \beta _2I_{\left\{ {\mathrm {Pair}} \right\}} + \beta _3I_{\left\{ {\mathrm {Order}} \right\}} + {\mathrm {Participant}}\,{\mathrm {ID}} + {\mathrm {Error}}$$

where *I*_{STAR}_ is an indicator variable for the condition level (for STAR and Audio), *I*_{Pair}_ is an indicator variable for the mentor–evaluator pair, and *I*_{Order}_ is an indicator variable for the order of the conditions (STAR–Audio and Audio–STAR). Participant ID denotes the random effects for the different participants. *β*_0_, *β*_1_, *β*_2_, and *β*_3_ are the regression coefficients of the model, and ${\mathrm {Error}}$ represents the residuals containing variance not explained by the model. The same model was applied to the Low First Responder Experience and Low Cric Experience subgroups. These separate analyses allowed to inspect the variance introduced by the participants’ years of experience or training level in cricothyroidotomies without directly including them as effects in the regression model.

For binary responses (CC-T), a logistic regression for binary responses was run^43^. Moreover, the proportional odds model was used to evaluate each of the subscores (i.e. ECP-1, to ECP-10, GRS-1 to GRS-6) independently^44^. The analysis of these binary and ordinal responses followed the same effects formulation as the previously described linear mixed regression model. Finally, the models’ performance and validity were evaluated using regression diagnostics (QQ plots, residual plots, and histograms). The tests confirmed that there was no assumption violation in the models (e.g. normality, independence and constant variance).

The linear mixed model revealed that both variables (mentor–evaluator pair and treatment order) had significant effects in the model. Specifically, both variables were significant (*p* ≤ 0.03) for all metrics except Completion Time for the Overall Population and Low First Responder Experience groups. For the Low Cric Experience, however, the mentor–evaluator pair variable was significant (*p* ≤ 0.05) only for the ECP-T, EOR, and CC-T metrics, and the treatment order variable was not significant for any metric. These analyses indicated that participants’ scores increased when using STAR after Audio. Contrarily, participants’ scores decreased when using Audio after STAR.

### Reporting summary

Further information on research design is available in the Nature Research Reporting Summary linked to this article.

## Supplementary information

Supplementary-materials

Reporting Summary

## Data Availability

Due to ethical concerns, supporting data related to the participants cannot be made openly available. Further information about the data that support the findings of this study is available from the corresponding author upon reasonable request.

## Code availability

The apps used in this work are hosted in GitHub, together with the necessary installation instructions. Mentor System App: www.github.com/edkazar/MentorSystemUWPWebRTC/releases; Mentee System App: www.github.com/practisebody/STAR/releases.
