# Supplementary material for: Evaluation of an augmented reality platform for austere surgical telementoring: a randomized controlled crossover study in cricothyroidotomies
Source: NPJ Digit Med. 2020 May 21;3:75. doi: 10.1038/s41746-020-0284-9 (PMC7242344; doi:10.1038/s41746-020-0284-9)
Supplement: Supplementary file 1 — Supplementary-materials [file 41746_2020_284_MOESM1_ESM.pdf]

PROPOSED ASSESSMENT TOOLS CRICOTHYROIDOTOMY PROCEDURE

Based on the DA FORM 7595-2-10 from the U.S. Army Training and Doctrine Command,[1] and the "Emergency Surgical Airway Using the Cric-Key" skill sheet from the Tactical Combat Casualty Care Handbook.[2] Grading scale was adapted following "Preparing for Emergency: A Valid, Reliable Assessment Tool for Emergency Cricothyroidotomy Skills" by Jacob Melchiors et al.[3] Global Rating Scale (GRS) for Cricothyrotomy as found in Friedman et al.[4]

Evaluator: _____________

[1] United States Army Training and Doctrine Command. PERFORM A SURGICAL CRICOTHYROIDOTOMY. May 2009. https://armypubs.army.mil/pub/eforms/DR_a/pdf/DA%20FORM%207595-2-10.pdf. Accessed September 3, 2018.

[2] Center for Army Lessons Learned. *Tactical Combat Casualty Care Handbook*. Vol 5.; 2017. https://usacac.army.mil/sites/default/files/publications/17493.pdf.

[3] Melchiors J, Todsen T, Nilsson P, et al. Preparing for emergency: a valid, reliable assessment tool for emergency cricothyroidotomy skills. *Otolaryngol Neck Surg*. 2015;152(2):260–265.

[4] Friedman Z, You-Ten KE, Bould MD, Naik V. Teaching lifesaving procedures: the impact of model fidelity on acquisition and transfer of cricothyrotomy skills to performance on cadavers. *Anesth Analg*. 2008;107(5):1663–1669.

Cric 1: ☐ AUDIO ☐ STAR Participant ID: ___________

IDENTIFICATION AND PALPATION					
**Correctly identified and palpated key surface landmarks on the anterior neck and the cricothyroid membrane.**	Step not performed	Palpated insecurely and did not find the proper location	Palpated insecurely and did not find the proper location but corrected	Palpated insecurely but found the proper location	Palpated confidently and found the proper location
INCISION					
**While stabilizing the larynx, made a vertical incision through the skin directly over the cricothyroid membrane.**	Step not performed	Not centered over membrane not correct length	Not centered over membrane but correct length	centered over membrane but not correct length	Centered over membrane and correct length
**While continuing to stabilize the larynx, used tool or fingers to expose the cricothyroid membrane.**	Step not performed	Failed to maintain the opening of the skin incision	Clumsily maintained the opening (used tools clumsily)	Used tools with difficulties but corrected.	Used tool correctly to dilate opening. Maintain the opening correctly
**Used the scalpel to make a horizontal incision through the cricothyroid membrane.**	Step not performed	Incision performed unsuccessfully. Tool mishandled	Incision performed, but incorrectly. Tool mishandled	Incision performed successfully and correctly. Tool mishandled.	Incision performed successfully and correctly. Tool used correctly.
INSERT TUBE					
**Inserted the Crickit and Melker cannula through the cricothyroid membrane directed distally towards the lungs until the flange contacted the skin of the neck.**	Step not performed	Failed to insert the tube	Struggled during tube insertion	Struggled during tube insertion, but corrected	Correctly inserted tube
**Verbalized feeling for tracheal rings while inserting the Cric-Key**	Did not verbalized feeling for tracheal rings			Correctly verbalized feeling for tracheal rings	
**Removed the Cric-Key, leaving the Melker cannula in place.**	Did not remove Cric-Key		Did not remove Cric-Key, but corrected	Correctly removed Cric-Key	
**Inflated the cuff of the Melker cannula with 10 ml of air.**	Step not performed	Over-inflated the cuff	Did not inflate the cuff	Under-inflated the cuff	Inflated cuff correctly
**Checked for air exchange and verified placement of the tube by assessing for bilateral rise and fall of the chest.**	Step not performed	Failed to check tube placement with no chest rise	Air exchange not achieved even after verifying tube placement	Air exchanged achieved after verifying tube placement	Air exchanged achieved with correct tube placement
SECURE AND DRESSING					
**If air exchange was adequate, secured the Melker cannula in place.**	Step not performed	Attempted to secure tube, but failed	Struggled to secure the tube	Secured the tube but could improve	Secured the tube correctly

Cric 1:

GRS: Global Rating Scale for cricothyroidotomy

Preparation for Procedure	1	2	3	4	5
	Did not organize equipment well. Has to stop procedure frequently to prepare equipment.		Equipment generally organized. Occasionally has to stop and prepare items.		All equipment neatly organized prepared and ready for use
Respect for Tissue	1	2	3	4	5
	Frequently used unnecessary force on tissue or caused damage		Careful handling of tissue but occasionally caused inadvertent damage		Consistently handled tissues appropriately with minimal damage
Time and Motion	1	2	3	4	5
	Many unnecessary moves		Efficient time/motion but some unnecessary moves		Clear economy of movement and maximum efficiency
Instrument Handling	1	2	3	4	5
	Repeatedly makes tentative or awkward moves with instruments		Competent use of instruments but occasionally appeared stiff or awkward		Fluid moves with instruments and no awkwardness
Flow of Procedure	1	2	3	4	5
	Frequently stopped procedure and seemed unsure of next move		Demonstrated some forward planning with reasonable progression of procedure		Obviously planned course of procedure with effortless flow from one move to the next
Knowledge of Procedure	1	2	3	4	5
	Deficient knowledge		Knew all important steps of procedure		Demonstrated familiarity with all aspects of procedure

EOR: Evaluator's overall rating (1-100) ___________

≥ 90 Excellent I hope that this individual is on call if I am injured
80-89 This individual will be able to perform the exposure with minimal difficulty in an expeditious fashion.
70-79 The participant might need to look at a text to refresh their memory but will be able to perform the exposure
60-69 This participant could do the exposure with experienced help, but will struggle if left alone
<60 The patient has exsanguinated. Participant is not ready to perform the exposure.

The overall score should be the instructor's subjective rating of how well the surgeon performed. This will be compared to the objective score for validating the scoring method.

Critical Criteria:

Obtained a patent airway with the emergency surgical airway. **(Yes/No)**
Identified the location of the cricothyroid membrane. **(Yes/No)**
Performed procedure in a manner that was safe to the casualty. **(Yes/No)**

Cric 2: ☐ AUDIO ☐ STAR Participant ID: ______________

IDENTIFICATION AND PALPATION					
**Correctly identified and palpated key surface landmarks on the anterior neck and the cricothyroid membrane.**	Step not performed	Palpated insecurely and did not find the proper location	Palpated insecurely and did not find the proper location but corrected	Palpated insecurely but found the proper location	Palpated confidently and found the proper location
INCISION					
**While stabilizing the larynx, made a vertical incision through the skin directly over the cricothyroid membrane.**	Step not performed	Not centered over membrane not correct length	Not centered over membrane but correct length	centered over membrane but not correct length	Centered over membrane and correct length
**While continuing to stabilize the larynx, used tool or fingers to expose the cricothyroid membrane.**	Step not performed	Failed to maintain the opening of the skin incision	Clumsily maintained the opening (used tools clumsily)	Used tools with difficulties but corrected.	Used tool correctly to dilate opening. Maintain the opening correctly
**Used the scalpel to make a horizontal incision through the cricothyroid membrane.**	Step not performed	Incision performed unsuccessfully. Tool mishandled	Incision performed, but incorrectly. Tool mishandled	Incision performed successfully and correctly. Tool mishandled.	Incision performed successfully and correctly. Tool used correctly.
INSERT TUBE					
**Inserted the Crickit and Melker cannula through the cricothyroid membrane directed distally towards the lungs until the flange contacted the skin of the neck.**	Step not performed	Failed to insert the tube	Struggled during tube insertion	Struggled during tube insertion, but corrected	Correctly inserted tube
**Verbalized feeling for tracheal rings while inserting the Cric-Key**	Did not verbalized feeling for tracheal rings		Correctly verbalized feeling for tracheal rings		
**Removed the Cric-Key, leaving the Melker cannula in place.**	Did not remove Cric-Key		Did not remove Cric-Key, but corrected	Correctly removed Cric-Key	
**Inflated the cuff of the Melker cannula with 10 ml of air.**	Step not performed	Over-inflated the cuff	Did not inflate the cuff	Under-inflated the cuff	Inflated cuff correctly
**Checked for air exchange and verified placement of the tube by assessing for bilateral rise and fall of the chest.**	Step not performed	Failed to check tube placement with no chest rise	Air exchange not achieved even after verifying tube placement	Air exchanged achieved after verifying tube placement	Air exchanged achieved with correct tube placement
SECURE AND DRESSING					
**If air exchange was adequate, secured the Melker cannula in place.**	Step not performed	Attempted to secure tube, but failed	Struggled to secure the tube	Secured the tube but could improve	Secured the tube correctly

Cric 2:

GRS: Global Rating Scale for cricothyroidotomy

Preparation for Procedure	1	2	3	4	5
	Did not organize equipment well. Has to stop procedure frequently to prepare equipment.		Equipment generally organized. Occasionally has to stop and prepare items.		All equipment neatly organized prepared and ready for use
Respect for Tissue	1	2	3	4	5
	Frequently used unnecessary force on tissue or caused damage		Careful handling of tissue but occasionally caused inadvertent damage		Consistently handled tissues appropriately with minimal damage
Time and Motion	1	2	3	4	5
	Many unnecessary moves		Efficient time/motion but some unnecessary moves		Clear economy of movement and maximum efficiency
Instrument Handling	1	2	3	4	5
	Repeatedly makes tentative or awkward moves with instruments		Competent use of instruments but occasionally appeared stiff or awkward		Fluid moves with instruments and no awkwardness
Flow of Procedure	1	2	3	4	5
	Frequently stopped procedure and seemed unsure of next move		Demonstrated some forward planning with reasonable progression of procedure		Obviously planned course of procedure with effortless flow from one move to the next
Knowledge of Procedure	1	2	3	4	5
	Deficient knowledge		Knew all important steps of procedure		Demonstrated familiarity with all aspects of procedure

EOR: Evaluator's overall rating (1-100) ____________

≥ 90 Excellent I hope that this individual is on call if I am injured

80-89 This individual will be able to perform the exposure with minimal difficulty in an expeditious fashion.

70-79 The participant might need to look at a text to refresh their memory but will be able to perform the exposure

60-69 This participant could do the exposure with experienced help, but will struggle if left alone

<60 The patient has exsanguinated. Participant is not ready to perform the exposure.

The overall score should be the instructor's subjective rating of how well the surgeon performed. This will be compared to the objective score for validating the scoring method.

Critical Criteria:

Obtained a patent airway with the emergency surgical airway. **(Yes/No)**

Identified the location of the cricothyroid membrane. **(Yes/No)**

Performed procedure in a manner that was safe to the casualty. **(Yes/No)**
